# Physical Activity as a Predictor of Internet Gaming Disorder in a Swiss Male Cohort (C-SURF): L’activité physique comme prédicteur des troubles liés aux jeux vidéo en ligne dans une cohorte de jeunes hommes suisses (C-SURF)

**DOI:** 10.1177/07067437241293979

**Published:** 2024-11-08

**Authors:** Adam Nowotarski, Stephane Rothen, Filip Kasina, Daniele Zullino, Gabriel Thorens

**Affiliations:** 27230 Service d'Addictologie, HUG, Geneve, Switzerland

**Keywords:** internet use disorder, behavioral addictions, behavior problems, Usage problématique d’internet, addictions comportementales, troubles de comportement

## Abstract

**History and Objectives:**

This study addresses the increasing global concerns surrounding Internet gaming disorder (IGD) by exploring their connection to physical activity (PA) as a potential preventive and early intervention measure. The research aims to examine the relationship between PA and the progression of IGD.

**Methods:**

Longitudinal data from the Cohort research on Substance Use Risk Factors involving young Swiss men undergoing army conscription was employed. PA levels were assessed using the International PA Questionnaire (IPAQ), while the Game Addiction Scale (GAS) and Compulsive Internet Use Scale determined IGD presence. Analysis involved zero-inflated negative binomial regression models.

**Results:**

Higher PA levels were associated with lower IGD risk. Notably, individuals engaging in high physical exercise exhibited a lower IGD prevalence compared to those with moderate or low activity levels.

**Discussion:**

The study suggests that intensive physical exercise might serve as a preventive strategy against developing IGD. This protective effect could stem from various mechanisms. However, the study's limitations, such as a male-only sample and a small low-activity group, should be considered when interpreting results.

**Conclusion:**

The longitudinal study demonstrates the positive influence of intense physical exercise on mitigating gaming-related issues. These findings underscore the potential of PA interventions in addressing the growing problem of IGDs and their impact on health. Further research is necessary to uncover underlying mechanisms behind the PA–IGD relationship and validate these findings across diverse demographics.

## Résumé

**Contexte et objectifs:**

La présente étude répond aux préoccupations mondiales croissantes concernant les troubles liés aux jeux vidéo en ligne en explorant le lien de ces troubles avec l’activité physique afin d’en faire une mesure potentielle de prévention et d’intervention précoce. Notre recherche vise à examiner la relation entre l’activité physique et l’évolution des troubles liés aux jeux vidéo en ligne.

**Méthodes:**

Les données longitudinales de l’étude de cohorte sur les facteurs de risque liés à la consommation de substances psychoactives (Cohort Study on Substance Use Risk Factors [C-SURF]), à laquelle ont participé de jeunes Suisses enrôlés dans l’armée, ont été utilisées. Les niveaux d’activité physique ont été évalués à l’aide de l’International Physical Activity Questionnaire (IPAQ), tandis que la Game Addiction Scale (GAS) et la Compulsive Internet Use Scale (CIUS) ont servi à déterminer la présence d’un trouble lié aux jeux vidéo en ligne. L’analyse a fait appel à des modèles de régression binomiale négative gonflés à zéro.

**Résultats:**

Des niveaux d’activité physique plus élevés étaient associés à un risque plus faible de développer un trouble lié aux jeux vidéo en ligne. Notamment, les personnes qui font beaucoup d’activité physique présentaient une prévalence plus faible de trouble lié aux jeux vidéo en ligne que celles ayant un niveau d’activité modéré ou faible.

**Discussion:**

Notre étude indique que l’exercice physique intensif pourrait servir de stratégie préventive contre le développement du trouble lié aux jeux vidéo en ligne. Cet effet protecteur pourrait provenir de divers mécanismes. Cependant, les limites de l’étude, comme le fait que l’échantillon soit exclusivement masculin et que le groupe pratiquant peu d’activité physique soit peu nombreux, doivent être prises en compte lors de l’interprétation des résultats.

**Conclusion:**

L’étude longitudinale montre l’influence positive de l’exercice physique intense sur l’atténuation des problèmes liés aux jeux vidéo. Ces résultats soulignent le potentiel des interventions axées sur l’activité physique pour répondre au problème croissant des troubles liés aux jeux vidéo en ligne et à leur impact sur la santé. Des recherches supplémentaires sont nécessaires pour découvrir les mécanismes sous-jacents de la relation entre activité physique et troubles liés aux jeux vidéo en ligne et pour valider ces résultats dans divers groupes démographiques.

## Introduction

Online gaming's popularity has surged recently, leading to a range of problems related to excessive gaming.^1^

In May 2013, Internet Gaming Disorder (IGD) was added to the *Diagnostic and Statistical Manual of Mental Disorders-IV (DSM-5)*, marking the first formal recognition of gaming as a mental health disorder (American Psychiatric Association, 2013).^2^

The *DSM-5* specifies nine criteria for diagnosing IGD, requiring at least five of them to be present for at least 12 months.^3^

The World Health Organization defines IGD in the ICD-11 as a pattern of gaming behavior where control diminishes, gaming takes precedence over other activities, and negative consequences persist for at least 12 months (WHO). The prevalence of online gaming problems varies but is notably high among youth, ranging from 1% to 10% in European and North American studies.^1^

IGD is thought to be particularly prevalent amongst youths, and in many Asian countries, rates among this population are thought to be as high as 10–15% and 1–10% in their counterparts in other occidental countries.^1^

The socioeconomic loss due to excessive Internet use in the Republic of Korea was estimated at between 1.5 and 4.5 billion U.S. dollars in 2009.^4^

In Korea, “Internet addiction” mostly from gaming has been identified as the largest health problem experienced by young people.^5^

Reducing the impact of a particular health condition, both on the broader public and individuals’ health, is crucial. Identifying predictive factors aids in pinpointing at-risk populations and providing timely preventive interventions.

It has been shown that IGD could be related to maladaptive comportment such as substance use or other behavioral addictions as gambling disorder. Studies have shown similarities in risk factors, personality traits, neurobiological manifestations and symptomatology.^6^

In contrast, physical activity (PA) has positive effects on health, including improved feelings of self-confidence, self- accomplishment, self-worth, mood, and social relationships.^7^

It is therefore hypothesized that PA competes with IGD for these benefits, acting as a protective behavior against IGD development.^8^

With IGD's prevalence continuously increasing, there has been an escalation in its interest in the research community. Despite an increasing number of cross-sectional studies, there is a paucity of longitudinal studies examining risk and protective factors especially of those whose aim is to determine PA as a predictive factor for IGD.^9^

Our primary objective is to assess the potential association between PA and IGD, leveraging data from the Comprehensive Substance Use and Risk Factors (C-SURF) database, available at https://www.c-surf.ch. The C-SURF database encompasses a longitudinal survey, which scrutinized various elements including substance consumption and problematic behaviors, often categorized as behavioral addictions. This dataset specifically focuses on male individuals who presented themselves at military recruitment centers situated in the Swiss municipalities of Lausanne and Windisch.

This study relies on data obtained from three distinct survey waves, conducted in the years 2010, 2012, and 2016. The central aim of this investigation is to ascertain the presence of a potential correlation among several key variables, including self-reported PA, as evaluated by the International PA Questionnaire (IPAQ), self-reported compulsive internet use, as assessed by the Compulsive Internet Use Scale (CIUS), and self-reported IGD, as quantified by the Game Addiction Scale (GAS).

## Methods

### Study Design

The C-SURF project tracks drug use in young Swiss adults over a minimum of 10 years. A representative sample of Swiss young men, encompassing approximately 98% of all Swiss male recruits were invited to participate in C-SURF. Consenters completed online questionnaires in either French or German, reaching the majority of French and a sizable portion of German-speaking Swiss youth due to the centers’ geographic coverage.

Strict confidentiality measures were in place throughout the study, with personal data and responses secured using anonymous coding. Only project coordinators had access to participants’ identities, ensuring data privacy. Importantly, participation or nonparticipation in the study had no bearing on the army recruitment process.

Approval for the study was granted by the local Ethics Committee (Protocol No. 15/07). All participants gave written informed consent to their participation in the study.

### Participants

A total of 6,378 people were invited to participate. Among them, 5,987 (93.9%) participants completed the questionnaires at baseline and of these, 5,125 (85.6%) completed the follow-up questionnaires for which they were rewarded 50 Chf. The mean delay between baseline and follow-up measurements was 65.7 (SD 5.1) months. The mean age was 20.0 (SD 1.2) years old at baseline and 25.4 (SD 1.3) years old at follow-up. Missing values were imputed in specific situations (see below) and the remaining missing values were deleted list wise leading to a final sample of 5,050 participants (98.5% of follow-up responders) that was analyzed. Demographics are summarized in Table 1.

**Table 1.** Sample Description.

Characteristic	Mean (SD)/*n* (%)
Age	
At baseline	20.0 (1.2)
At follow up	25.4 (1.3)
Language	
French	2869 (56%)
German	2256 (44%)
Education	
Elementary	3681 (73.6%)
Secondary	1245 (24.9%)
University	78 (1.6%)
Physical activity • Low	290 (5.7%)
Moderate	1231 (24.4%)
High	3529 (69.9%)
CIUS^a^	9.2 (8.9)
**GAS^b^**	**3.2 (4.2)**

^a^ Compulsive Internet Use Scale.

^b^ Game Addiction Scale.

### Measurements

#### International Physical Activity Questionnaire

The IPAQ is a standardized questionnaire that is used to assess PA levels in adults aged 18 to 65 years.

It is an open access questionnaire used as an indicator of PA, especially in large population studies or in the context of PA surveillance.

The version used in this study is the seven-question short form. It is important to remember that the IPAQ relies on self-reported data, which might be skewed by social desirability bias and recall bias. The IPAQ short form asks about three specific types of activity undertaken in the four following domains: leisure time PA, domestic and gardening activities, work-related PA and transport-related PA. The specific types of activity assessed are walking, moderate-intensity activities and vigorous-intensity activities. The items in the short IPAQ form were structured to provide separate scores on walking, moderate-intensity and vigorous-intensity activity. Computation of the total score for the short form requires summation of the duration (in minutes) and frequency (days) times a coefficient of 3.3 for walking, 4.0 for moderate-intensity and 8.0 for vigorous-intensity activities. Based on these scores three categories of PA were created: Low, Moderate and High.

One way to calculate one's energy expenditure is using metabolic equivalents, also known as METs. First, a continuous score defined as MET-min per week was created as follows: MET level × minutes of activity × events per week.

Category 1 represents the lowest level of PA. The individuals who do not meet the criteria for categories 2 or 3 are considered low/inactive.

Category 2 is composed of any one of the following three criteria:

- Three or more days of vigorous activity of at least 20 min per day
- Five or more days of moderate-intensity activity
- Five or more days of any combination of walking, moderate-intensity or vigorous intensity activities achieving a minimum of at least 600 MET-min/week

Category 3 is composed of either of the following two criteria:

- Vigorous-intensity activity on at least 3 days and accumulating at least 1,500 MET-minutes/week
- Seven or more days of any combination of walking, moderate-intensity or vigorous intensity activities achieving a minimum of at least 3,000 MET-minutes/week

#### Compulsive Internet Use Scale

The CIUS is a 14-Likert-scaled questionnaire that is based on the *DSM-IV* criteria for substance abuse and pathological gambling.

The scale includes typical symptoms of compulsive Internet use such as the inability to control Internet use, mental and behavioral preoccupation with online activities, agitation associated with the inability to go online, mood change, and conflicts with significant others over Internet use.^10^

The tool demonstrated high factorial stability across time, across several samples, and across subsamples. It shows high correlations with concomitant and criteria variables and high internal consistency, supporting its validity and reliability.^11^

The CIUS was used to screen for the presence of compulsive Internet use. Total score was computed using the sum of the 14 items giving a range of possible scores between 0 and 56. In this sample, reliability measured with Cronbach alpha was good (0.92).

#### Game Addiction Scale

The 7-item GAS is a scale developed by Lemmens et al. that is designed based on the *DSM-IV* criteria for Dependence and Pathological Gambling.^12^

The GAS consists of seven Likert-type questions, each of which starts with the question “Over the last six months, how often…” (1 = *never*, 2 = *rarely*, 3 = *sometimes*, 4 = *often*, and 5 = *very often*). For instance, “How often did you consider playing a game all day long over the past 6 months?” The GAS's overall score ranges from 7 to 35, with higher scores suggesting greater levels of gaming addiction.^3^

The GAS was used to screen for the presence of IGD. The total score was computed using the sum of the seven items giving a range of possible scores between 0 and 28. In this sample, reliability measured with Cronbach alpha was good (0.84).

#### Skipping Questions

Skipping questions determine whether the respondent has participated or not in the said activity and in this case allowed for the creation of Yes–No variables for gaming and compulsive internet use. Those that responded “No” simply skipped the section and didn't complete the corresponding questionnaire. Therefore, a score of 0 was assigned to the CIUS (*n* = 266, 5.2%) or the GAS (*n* = 868, 16.9%) in order to not exclude participants who did not play games or use the internet at follow-up but are informative. A person-mean imputation was carried out for the two scales mentioned above for subjects who indicated Yes at the skipping question and with only one missing item (CIUS: *n* = 16; GAS: *n* = 3). Subjects who had more than one item on a particular scale (CIUS: *n* = 4; GAS: *n* = 5) missing were excluded from the sample.

### Statistical Analysis

The two total scores (CIUS and GAS) were checked for normality prior to analysis. They all appeared to be positively skewed with an excess number of zeros, some by design (due to the skipping question) and some because subjects really answered negatively to each item. In order to take into account the excess of zeros and the positively skewed scores different models were tested and the ones which fitted best the data was retained. In order to achieve this goal, the fit of a Poisson, quasi-Poisson, negative binomial, hurdle Poisson, hurdle negative binomial, zero-inflated Poisson, and zero-inflated negative binomial model were compared with a likelihood-ratio test or the equivalent when necessary. The best fit was found for the zero-inflated negative binomial regression model. Two zero-inflated negative binomial regression was therefore performed. Age at baseline, language (reference level was French-speaking), and PA were the independent variables (reference level was low activity), while the dependent variables were the CIUS score for the first regression and the GAS score for the second. The output of zero-inflated negative binomial models comprises two components. The first one is a negative binomial count model whose estimates can be interpreted as the strength of the relationship between the independent variables and the dependent variable. The second component is a classical logistic regression (binomial model) which indicates the probability of answering 0 to the dependent variable.

Since 862 (14.4%) subjects didn’t participate in the follow-up, sensitivity analysis has been also performed as follow: the two zero-inflated negative binomial regressions were done on 29 imputed samples complete samples (*n* = 5987) created by multiple imputations (twice the percentage of follow up-up nonparticipants) using fully conditional specification.^13^

## Results

### Compulsive Internet Use Scale

Table 2 (see Supplemental Table S2A and S2B) shows the results of the CIUS analysis. Regarding the binomial part of the model, the likelihood of having a score of zero on the CIUS is 1.23 (CI [1.01, 1.49], *p* <0.05) times higher in the German speaking subjects compared to the French-speaking subjects. The likelihood of having a score of zero on the CIUS is 1.92 (CI [1.47, 2.50], *p* <0.001) times higher in the subjects having high PA compared to the subjects having moderate PA. The categories of age, intensity: moderate versus low and intensity: high versus low are not statistically significant (*p* >5%). Regarding the count part, the German speaking subjects compared to the French speaking subjects have a lower score (RR: 0.87, CI [0.83, 0.92], *p* <0.001). The subjects with a high PA have a lower CIUS score compared to the subjects with low PA (RR: 0.80, CI [0.71, 0.89], *p* < 0.001). The subjects with a high PA have a lower CIUS score compared to the subjects with moderate PA (RR 0.87, CI [0.82, 0.93], *p* <0.001). The categories of age and intensity: moderate versus low are not statistically significant (*p* >5%). Sensitivity analysis (Supplemental Table S2a) provides the same conclusions.

**Table 2.** Results for the Zero-Inflated Negative-Binomial Regression for the Compulsive Internet Use Scale (CIUS).

Variables	Count part rate ratio [95% CI]	Zero part odds ratio [95% CI]
Intercept	11.85*** [7.56, 18.59]	
Age	1.01 [0.98, 1.03]	1.07 [1.00, 1.16]
German-speaking (vs. French-speaking)	0.87*** [0.83, 0.92]	1.23* [1.01;1.49]
Physical activity: Moderate (vs. low)	0.91 [0.81, 1.03]	0.79 [0.48, 1.29]
Physical activity: High (vs. low)	0.80*** [0.71, 0.89]	1.51 [0.97, 2.35]
Physical activity: High (vs. moderate)—post-hoc	0.87*** [0.82, 0.93]	1.92*** [1.47, 2.50]

### Game Addiction Scale

Table 3 shows the results for the GAS analysis. Regarding the binomial part of the model, the likelihood of having a score of zero on the GAS is 1.59, (CI [1.33, 1.90], *p* <0.001) times higher in the German speaking subjects compared to the French speaking subjects. The likelihood of having a score of zero on the GAS is 1.29 (CI [1.05, 1.59], *p* <0.005) times higher in the subjects having high PA compared to the subjects having moderate PA. The categories of age, intensity: moderate versus low and intensity: high versus low are not statistically significant (*p* >5%). Regarding the count part, the German-speaking subjects compared to the French speaking subjects have a lower score RR: 0.93 (CI [0.86, 1.00], *p* <0.005). The subjects with a high PA have a lower GAS score compared to the subjects with moderate PA RR 0.90 (CI [0.83, 0.98], *p* <0.005). The categories of age and intensity: moderate versus low and intensity: high versus low are not statistically significant (*p* >5%). Sensitivity analysis (Supplemental Table S2b) provides the same conclusions.

**Table 3.** Results for the Zero-Inflated Negative-Binomial Regression for the Gaming Addiction Scale (GAS).

Variables	Count (rate ratio)	Zero (OR)
Intercept	3.30*** [1.74, 6.26]	
Age	1.02 [0.99, 1.05]	1.02 [0.95, 1.09]
German-speaking (vs. French-speaking)	0.93* [0.86, 1.00]	1.59*** [1.33, 1.90]
Physical activity: Moderate (vs. low)	0.96 [0.82, 1.00]	1.08 [0.70, 1.66]
Physical activity: High (vs. low)	0.87 [0.75, 1.01]	1.40 [0.93, 2.08]
Physical activity: High (vs. moderate)—post-hoc	0.90* [0.83, 0.98]	1.29* [1.05, 1.59]

## Discussion

In the present study, the data shows that PA regarded as being high versus moderate is related to a smaller prevalence of IGD. One hypothesis could be that the subjects who do intensive PA have less time on their hands and in result may have less time to spend on their phones/ the internet. Considering the criteria of intensive PA (vigorous-intensity activity on at least 3 days and accumulating at least 1,500 MET-minutes/week and/or 7 or more days of any combination of walking, moderate-intensity or vigorous intensity activities achieving a minimum of at least 3000 MET-minutes/week), one may consider these subjects being more health oriented and therefore less prone to developing IGD.

We have demonstrated above that IGD could be related to maladaptive behaviors such as substance use or gambling disorder. Studies have shown similarities in risk factors, personality traits, neurobiological manifestations, and symptomatology.^6^ In contrast, PA in itself may indirectly positively influence health behaviors such as overeating, smoking, alcohol consumption, substance abuse, stress management, risk taking, and others.^7^ It may be therefore concluded that intensive PA plays a role in diminishing IGD.

In addition to effects specific to intensive PA, several mechanisms of action (structured social events, general lifestyle changes, a nonconsumption social environment) have been discussed in the literature^14^ and could play a crucial role in the diminishing of IGD in the subjects who partake in intensive PA.

### Limitations

The data used in the present study can be considered representative of the Swiss male population. Nevertheless, there are some limitations, the most obvious of which is that the sample is limited to males who presented themselves at military recruitment centers for their military duty and therefore the test results are not fully generalizable.

We have a small group of people reporting low PA compared to the number of subjects reporting medium intensity and high intensity PA. This could in turn affect the results and explain the statistical nonsignificance in the medium to low and high to low PA.

Another limitation we could consider is the usage of internet at baseline, a question which was introduced later on in the sample collection in a future wave. It would have been interesting if internet use at baseline predicts higher internet usage.

## Conclusion

The results of our longitudinal study conclude that intensive PA may have beneficial effects against the development of internet use disorder.

## Supplemental Material

sj-docx-1-cpa-10.1177_07067437241293979 - Supplemental material for Physical Activity as a Predictor of Internet Gaming Disorder in a Swiss Male Cohort (C-SURF) Supplemental material, sj-docx-1-cpa-10.1177_07067437241293979 for Physical Activity as a Predictor of Internet Gaming Disorder in a Swiss Male Cohort (C-SURF) by Adam Nowotarski, MD, Stephane Rothen, PhD, Filip Kasina, MD, Daniele Zullino, MD, and Gabriel Thorens, MD in The Canadian Journal of Psychiatry

## References

[1] Saunders JB Hao W Long J , et al. Gaming disorder: its delineation as an important condition for diagnosis, management, and prevention. J Behav Addict. 2017;6(3):271-279. 28816494 10.1556/2006.6.2017.039 PMC5700714

[2] King DL Delfabbro PH . The cognitive psychology of internet gaming disorder. Clin Psychol Rev. 2014;34(4):298-308. 24786896 10.1016/j.cpr.2014.03.006

[3] Liu Y Wang Q Jou M Wang B An Y Li Z . Psychometric properties and measurement invariance of the 7-item Game Addiction Scale (GAS) among Chinese college students. BMC Psychiatry. 2020;20(1):484. 33008339 10.1186/s12888-020-02830-7 PMC7531159

[4] Lee HK Kim HS Lee TJ . Cost-effect analysis on the introduction of online game shut down regulation. Ministry of Gender Equality & Family. 2011.

[5] Saunders JB Hao W Long J , et al. Gaming disorder: its delineation as an important condition for diagnosis, management, and prevention. J Behav Addict. 2017;6(3):271-279. 28816494 10.1556/2006.6.2017.039 PMC5700714

[6] Lemmens JS Valkenburg PM Peter J . Development and validation of a game addiction scale for adolescents. Media Psychol. 2009;12(1):77-95.

[7] Blair SN Jacobs DR Powell J E K . Relationships between exercise or physical activity and other health behaviors. Public Health Rep. 1985;100(2):172-180. 3920715 PMC1424740

[8] Spruit A Assink M van Vugt E van der Put C Stams GJ . The effects of physical activity interventions on psychosocial outcomes in adolescents: a meta-analytic review. Clin Psychol Rev. 2016;45:56-71. 27064552 10.1016/j.cpr.2016.03.006

[9] Liew LWL Stavropoulos V Adams BLM Burleigh TL Griffiths MD . Internet gaming disorder: the interplay between physical activity and user–avatar relationship. Behav Inf Technol. 2018;37(6):558-574.

[10] Milasauskiene E Burkauskas J Podlipskyte A , et al. Compulsive internet use scale: psychometric properties and associations with sleeping patterns, mental health, and well-being in Lithuanian medical students during the coronavirus disease 2019 pandemic. Front Psychol. 2021;12:685137. 34512443 10.3389/fpsyg.2021.685137 PMC8428172

[11] Meerkerk GJ Van Den Eijnden RJ Vermulst AA Garretsen HF . The compulsive internet use scale (CIUS): some psychometric properties. Cyberpsychol Behav. 2009;12(1):1-6. 19072079 10.1089/cpb.2008.0181

[12] Lemmens JS Valkenburg P Peter J. Development and validation of a game addiction scale for adolescents. Media Psychol. 2009:12:77-95.

[13] Liu Y De A . Multiple imputation by fully conditional specification for dealing with missing data in a large epidemiologic study. Int J Stat Med Res. 2015;4(3):287-295. 27429686 10.6000/1929-6029.2015.04.03.7 PMC4945131

[14] Read JP Brown RA . The role of physical exercise in alcoholism treatment and recovery. Professional Psychology: Research and Practice. 2003;34:49-56.

