# Supplementary material for: Physical Activity as a Predictor of Internet Gaming Disorder in a Swiss Male Cohort (C-SURF): L’activité physique comme prédicteur des troubles liés aux jeux vidéo en ligne dans une cohorte de jeunes hommes suisses (C-SURF)
Source: Can J Psychiatry. 2024 Nov 8;69(12):855–60. doi: 10.1177/07067437241293979 (PMC11562881; doi:10.1177/07067437241293979)
Supplement: sj-docx-1-cpa-10.1177_07067437241293979 - Supplemental material for Physical Activity as a Predictor of Internet Gaming Disorder in a Swiss Male Cohort (C-SURF) [file sj-docx-1-cpa-10.1177_07067437241293979.docx]

Supplemental material

| **Variables** | **Count** | **Zero (OR)** |
| --- | --- | --- |
| **Intercept** | 11.52*** [7.47;17.76] | 0.04*** [0.01;0.18] |
| **Age** | 1.01 [0.99;1.03] | 1.06 [0.99;1.14] |
| **German-speaking (vs. French-speaking)** | 0.89*** [0.85;0.94] | 1.19* [0.99;1.44] |
| **Physical activity: Moderate (vs. Low)** | 0.93 [0.83;1.04] | 0.79 [0.51;1.24] |
| **Physical activity: High (vs. Low)** | 0.83*** [0.74;0.92] | 1.36 [0.91;2.04] |

Table 2a: Results from the zero-inflated negative binomial regression on the complete sample with multiple imputation (CIUS)

| **Variables** | **Count** | **Zero (OR)** |
| --- | --- | --- |
| **Intercept** | 3.54*** [1.92;6.52] | 0.17** [0.04;0.76] |
| **Age** | 1.02 [0.99;1.05] | 1.02 [0.95;1.09] |
| **German-speaking (vs. French-speaking)** | 0.93* [0.87;1.00] | 1.5*** [1.27;1.78] |
| **Physical activity: Moderate (vs. Low)** | 0.97 [0.83;1.12] | 1.04 [0.70;1.55] |
| **Physical activity: High (vs. Low)** | 0.89 [0.77;1.02] | 1.30 [0.90;1.88] |

Table 2b: Results from the zero-inflate negative binomial regression on the complete sample with multiple imputation (GAS)
